# Learning from the unexpected in life and DNA self-assembly

**DOI:** 10.3762/bjoc.11.292

**Published:** 2015-12-23

**Authors:** Jennifer M Heemstra

**Affiliations:** 1 Department of Chemistry and the Center for Cell and Genome Science, University of Utah, 315 S 1400 E, Salt Lake City, Utah 84112, United States

**Keywords:** aptamers, DNA self-assembly, nucleic acids

## Abstract

The greatest lessons in life and science often arise from the unexpected. Thus, rather than viewing these experiences as hindering our progress, they should be embraced and appreciated for their ability to lead to new discoveries. In this perspective, I will discuss the unexpected events that have shaped my career path and the early stages of my independent research program.

## Introduction

“Jennifer is not skilled at science.” This judgment was delivered upon me at the age of fourteen by my eighth grade science teacher, and was part of a recommendation that I not be placed on the science-intensive track of study as I entered high school. The extent to which this recommendation limited my access to science courses should have effectively ended any chance of my going on to pursue a career in this field. However, this event instead allowed me to view science as a fun hobby instead of a required subject, which led me to discover my love of science and began to pave the road to my future career in chemistry. At every step along this road, I benefited from fantastic mentors who shared with me their enthusiasm for life and learning. I also grew to recognize my love of supramolecular chemistry, and this has been the constant theme driving my research choices throughout my career. Much like a negative judgment about my aptitude for science unexpectedly led me to a career in chemistry, my lab has found that our greatest insights into the molecular recognition and self-assembly properties of DNA have come from unexpected results or failed experiments.

## Review

Entering high school, I knew that I enjoyed math, but was unsure of my career goals. Looking for a way to stay entertained after school, my friends suggested that I join them on the Science Olympiad team. My response was that this was obviously a terrible idea, as I was “not skilled at science.” They thankfully convinced me to join despite this, and in Science Olympiad, I found my first true mentor, Dr. Marcia Sprang. Dr. Sprang taught the advanced Chemistry and Physics courses at my high school, and also coached the Science Olympiad team. Over the four years that I was involved in Science Olympiad, Dr. Sprang convinced me that you don’t need “skill” to be a scientist, but rather you just need a love of learning and a passion for discovery. During this time, she also served as a model for the type of mentor that I would later hope to become – providing consistent encouragement and wisdom, while teaching students that through hard work, they can achieve things that they never thought possible. Reflecting on this story, I have only recently come to appreciate that had I been labeled “skilled at science,” I would have felt tremendous pressure to excel in this area, which likely would have killed my enthusiasm for the subject. However, being told that I had no natural aptitude for the subject removed this pressure, allowing me the freedom to pursue science for the pure enjoyment of learning.

Entering college at the University of California, Irvine, I brought with me the love of science instilled by my experiences in Science Olympiad and the mentoring of Dr. Sprang. However, I was still unsure which area of science I wanted to pursue. I began my college career convinced that I wanted to major in Biology, but quickly realized that this was not the right fit. As I found myself adrift and trying to formulate a new career plan, I decided to dispatch with the required Organic Chemistry courses, which were almost universally dreaded by my fellow Biology majors. I expected these courses to be difficult and frustrating, but instead found that learning about organic molecules and solving the complex puzzle of multi-step synthesis was fun and gratifying. Around this same time, I decided to begin working towards my honors thesis, which required me to find a lab where I could do research. Considering the amount of fun I had in my Organic Chemistry course, I thought that it would be interesting to experience research in a chemistry lab. I read through all of the faculty research descriptions, and while I was still years away from recognizing an underlying love of supramolecular chemistry, I was immediately drawn to the work of Prof. James Nowick, whose lab was focused on constructing and studying artificial β-sheet structures.

I am thankful that James provided me the opportunity to join his lab, as it was this experience that brought me from thinking of chemistry as a “fun course that I took” to something that I might want to spend my life pursuing. Even though I was just an undergraduate student, James (and my postdoctoral mentor, Dr. Mark Wilson) provided me with a significant amount of freedom in the lab. The ability to formulate a hypothesis, design experiments, and then test whether or not my ideas would work afforded me a sense of satisfaction that was entirely new and utterly intoxicating. This experience not only convinced me that I wanted to pursue a Ph.D. in Chemistry, but also continues to influence my mentoring style in my independent career. Specifically, I recognize that what convinced me I wanted a career in research was the freedom and autonomy of asking and answering my own questions, even just in the initial context of troubleshooting a project that had been designed for me. As a result, I not only have a great enthusiasm for mentoring undergraduate students in my own lab, but I place a high value on giving each student their own project, or a distinct piece of a larger project, so that they also can experience this joy of autonomy. As I began to ponder the next stage of my career, I recognized a second, very important lesson that I had learned from James – the importance of working for wonderful people. While the process of discovery was what had made me fall in love with research, having an advisor who was enthusiastic and supportive was what made it fun to come into lab each day. Even more importantly, I recognized that even though I was only an undergraduate researcher, James would be an ally and supporter throughout my career.

By the time that I was choosing a graduate program, I had learned the phrase “supramolecular chemistry” and recognized my excitement for the process of designing, building, and studying functional molecular architectures. In the process of researching graduate programs, I read a series of papers from the lab of Prof. Jeff Moore at the University of Illinois describing their pioneering work on helical phenylene ethynylene foldamers. As with many things in my scientific career, I was drawn to this work because it was “just *so* cool.” While I was still an undergrad, James offered to introduce me to Jeff by email, and this started a series of conversations about exciting future project ideas. I also came to realize that Jeff was exactly the type of mentor I hoped to find in graduate school, as he was a genuinely kind person, providing encouragement to the students in the lab, but also providing them the freedom to develop into independent scientists. On my first day in Jeff’s lab, I was given the freedom to choose which new idea I wanted to pursue, and every day after that, I was given the freedom to stumble, make mistakes, figure things out for myself, and ultimately “learn how to learn.” This process that Jeff fostered in his lab now forms the core of my mentoring philosophy. The part of my job I enjoy most is discussing research (and life in general) with the students and postdocs in my lab, especially since I am fortunate to have a research group that is filled with creative, motivated, and independent-minded scientists. Inspired by my time in the Moore Group, a large fraction of these conversations end with me saying something along the lines of “there must be a way to do this, but I don’t know exactly how – you get to go and figure it out.” My proudest moments are when one of my group members has taken on one of these challenges, mastered things that I have no idea how to do, and then they spend our lab meetings teaching these new concepts and ideas to me and the rest of the lab. In my opinion, achieving this level of independent learning and thinking is the pinnacle of success in a Ph.D. career.

During my Ph.D. studies, I had tremendous fun designing and studying organic foldamers, but I also began to be attracted to the especially privileged molecular recognition and self-assembly properties of nucleic acids. I think that Jeff actually recognized this before I did, as he chose the additional faculty members on my thesis committee to be Profs. Steve Zimmerman and Scott Silverman, who are both leaders in the field of nucleic acid molecular recognition. As my graduation neared, Jeff, Steve, and Scott all enthusiastically encouraged me to pursue postdoctoral research and then a career in academia. After a brief detour into industry while I waited for my husband to finish his Ph.D., I was fortunate to have the opportunity to join the lab of Prof. David Liu at Harvard University. Considering that I had made an early departure from my Biology major as an undergrad, I now found myself working in a chemical biology lab with almost no formal training in molecular or cellular biology. Fortunately, David provided me the freedom to make mistakes and learn from those around me, which allowed me to grow in my knowledge of this field that was almost entirely new to me. Additionally, David and my other former advisors provided me with critical mentoring as I went through the process of applying, interviewing, and negotiating for an academic position. This experience further reinforced to me the value of having great people on your side as you strike out into the world on your own.

Looking back on my early career, it is clear that gender and work-life balance issues have in many ways shaped the path that I have taken. I have expressed my perspective on some of these issues in depth elsewhere [1], and thus will only mention the topic briefly here. The data show that there is significant progress that still needs to be made to increase diversity and accommodate work-life balance in the sciences [2], and arguably much of this change needs to be wrought at the institutional level. However, I hope that my story demonstrates that every individual can make a tremendous impact in the areas of diversity and equity through the mentorship and advocacy that they provide to others.

As the students in my own lab near graduation and start to consider their next move, I encourage them to choose the place where they know they will thrive, both scientifically and personally. Not surprisingly, choosing to work for a wonderful mentor or at a company with supportive management is a central part of this advice. I know that I would not be where I am without the mentors who continue to support, encourage, and serve as role models for me in my independent career. I am honored to now have the opportunity to pay that debt forward by serving as a mentor to students and postdocs in my own lab, and it is my great hope that as these individuals go out from my lab, they will propagate this legacy of positive mentoring as they themselves move into positions of leadership.

**Figure 1:**
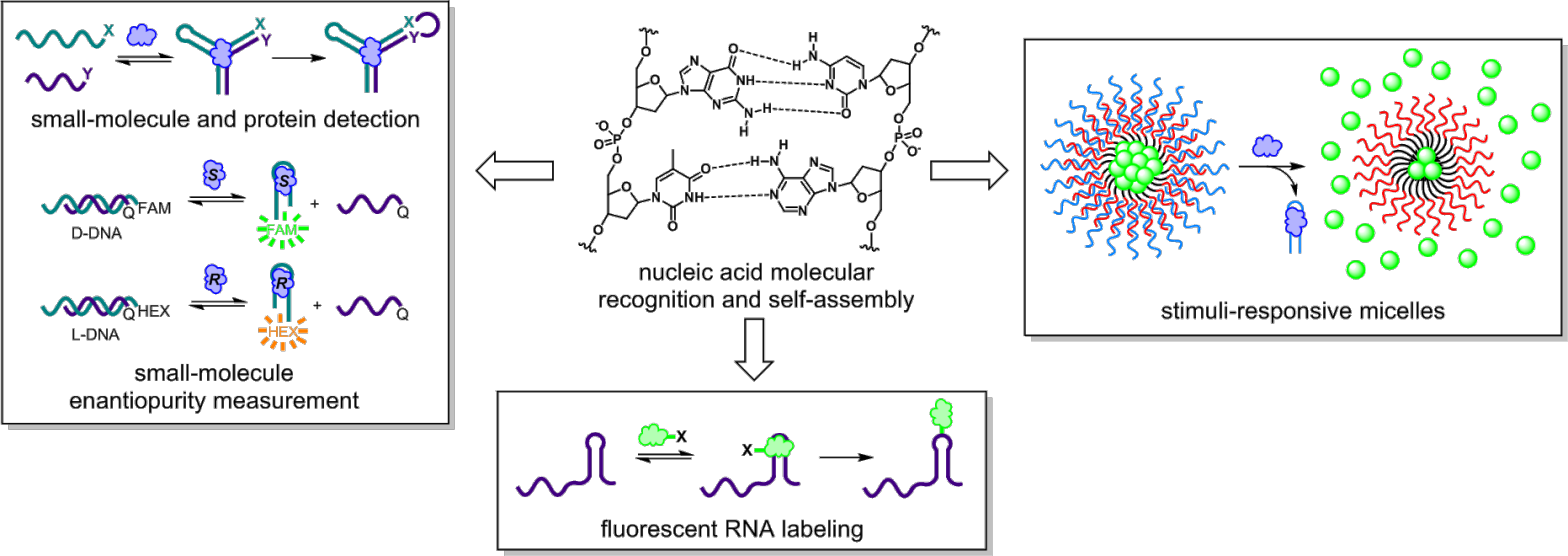
The common thread that is woven throughout our research program is the utilization of nucleic acid molecular recognition and self-assembly to generate functional architectures for biosensing and bioimaging. Adapted with permission from [3]. Copyright (2014) American Chemical Society. Adapted with permission from [4]. Copyright (2015) American Chemical Society.

In starting my independent career at the University of Utah, I never intentionally made a decision to work in a specific area of science. Instead, I brainstormed to generate a series of ideas, then narrowed down my list to the few that I was most excited about. This ended up being an interesting process, as I was able to look at the result and gain insight into who I was as a scientist, at least at that moment in my career. All of the project ideas that made the final cut involved using a combination of molecular recognition, self-assembly, and nucleic acids to build functional architectures for applications in biosensing or bioimaging (Figure 1). As my research group translated these (and many of their own) ideas into actual experiments, we began to recognize a paradigm in which our projects are designed with an application in mind, as we want to make things that will benefit society. However, while we are excited when one of our ideas works as planned, some of our greatest moments are when something doesn’t work for an unexpected reason. These “failed” experiments, while initially frustrating, have frequently led us to learn something new about the interactions of nucleic acids with each other or with ligands such as small molecules. These new insights are often the key to making a project successful, and sometimes even inspire the design a new project, but they can tend to get lost among all of the data in a manuscript, or relegated to the supporting information. Thus, in this perspective article, I am excited to have the opportunity to highlight some of these stories of unexpected results and what we learned from them.

During my initial brainstorming of project ideas, I was very drawn to the idea of working with DNA split aptamers. These recognition elements are comprised of two DNA (or RNA) sequences that selectively assemble in the presence of a small-molecule or protein target [5–6]. Thus, they combine my two favorite themes, as they use molecular recognition to drive self-assembly. At the point that we began our research program, all of the reported work on split aptamers had focused on detecting non-covalent assembly for systems at equilibrium [7]. Building upon this work, we were intrigued by the question of whether these DNA assembly events could be covalently trapped, which would allow the initial binding event to be “remembered” even if the target became unbound from the split aptamer. We reasoned that this might improve the sensitivity of split aptamer-based detection assays, and also that by converting the presence of a target molecule into the output of a DNA ligation event, we might be able to use split aptamers in assay formats that were previously inaccessible with these affinity reagents.

**Figure 2:**
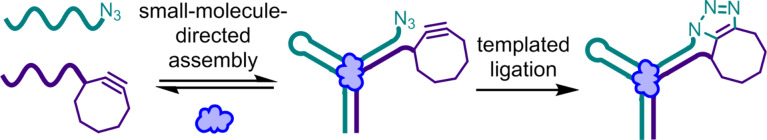
Split aptamers use molecular recognition to drive the assembly of two DNA strands. Placing reactive functional groups at the termini of the split aptamer fragments enables these assembly events to be covalently trapped. Reprinted with permission from [11]. Copyright (2011) American Chemical Society.

In our first attempt to demonstrate this principle of split aptamer ligation, we functionalized one fragment of the cocaine-binding DNA split aptamer [8] with a cyclooctyne and the other fragment with an azide. Although the cyclooctyne and azide are inherently reactive towards one another [9], we hypothesized that in the context of the free split aptamer fragments at low concentrations, the second order reaction would be relatively slow; however, addition of cocaine would drive assembly of the DNA strands, placing the two reactants in close proximity to one another and thus dramatically increasing the reaction rate (Figure 2). In our first experiment, we tested the hypothesis that the untemplated second order reaction would be sufficiently slow to prevent accumulation of the background signal. We were surprised to observe significant ligation between the split aptamer fragments, even in the absence of cocaine. While our initial thought was that this background was the result of a second order reaction between the functional groups on the DNA strands, this was quickly disproven, as we found that the untemplated ligation yield was not dependent upon DNA concentration. This led us to take a step back and think more critically about the fundamental principles behind split aptamer assembly. We quickly recognized that the function of split aptamers relies on a thermodynamic balancing act in which the enthalpic gain of base pairing and base stacking in the assembled state is weighed against the entropic cost of assembly. According to this logic, split aptamers will function best when the enthalpy for hybridization between the DNA strands is tuned such that in the absence of the ligand, this enthalpic gain is not quite sufficient to overcome the entropic cost of assembly. This poises the DNA strands at the brink of assembly, where the small amount of additional enthalpy gained through target binding can dramatically shift the equilibrium to favor the assembly of the split aptamer. To test this hypothesis, we carried out a simple experiment in which we measured the ligation yield for the two split aptamer fragments in the absence of cocaine, but in the presence of varying concentrations of sodium chloride, as increasing ionic strength increases the enthalpic gain for nucleic acid duplex formation [10]. We found that the ligation yield consistently increased with increasing ionic strength, which served as an initial validation of our hypothesis regarding the thermodynamics of split aptamer assembly. In the short term, this allowed us to overcome the challenge of background signal by reducing the ionic strength, and we were delighted to observe dose-dependent ligation of the split aptamer fragments with increasing concentrations of cocaine [11].

While the insight we gained into balancing the thermodynamics of split aptamer assembly seemed fairly straightforward, the lessons we learned from this unexpected experimental result played a critical role in our success with many of the experiments that soon followed. Having recognized that split aptamer assembly could be tuned much like the dial on a radio, we soon became enthralled by our ability to shift the equilibrium for this assembly process in predictable ways. As described above, this was initially achieved by simply changing the ionic strength of the solution. However, when we moved to experiments in biological fluids, where the ionic strength is difficult to change, we began to explore the tuning of enthalpy through rational mutations to the DNA sequences themselves. We were gratified to find that by adding or taking away base pairs, we could reliably tune the equilibrium for assembly of the split aptamer fragments. This provided us with the power to adapt the cocaine split aptamer to function with optimal signal-to-background in higher ionic strength samples such as human blood serum and artificial urine media [12].

**Figure 3:**
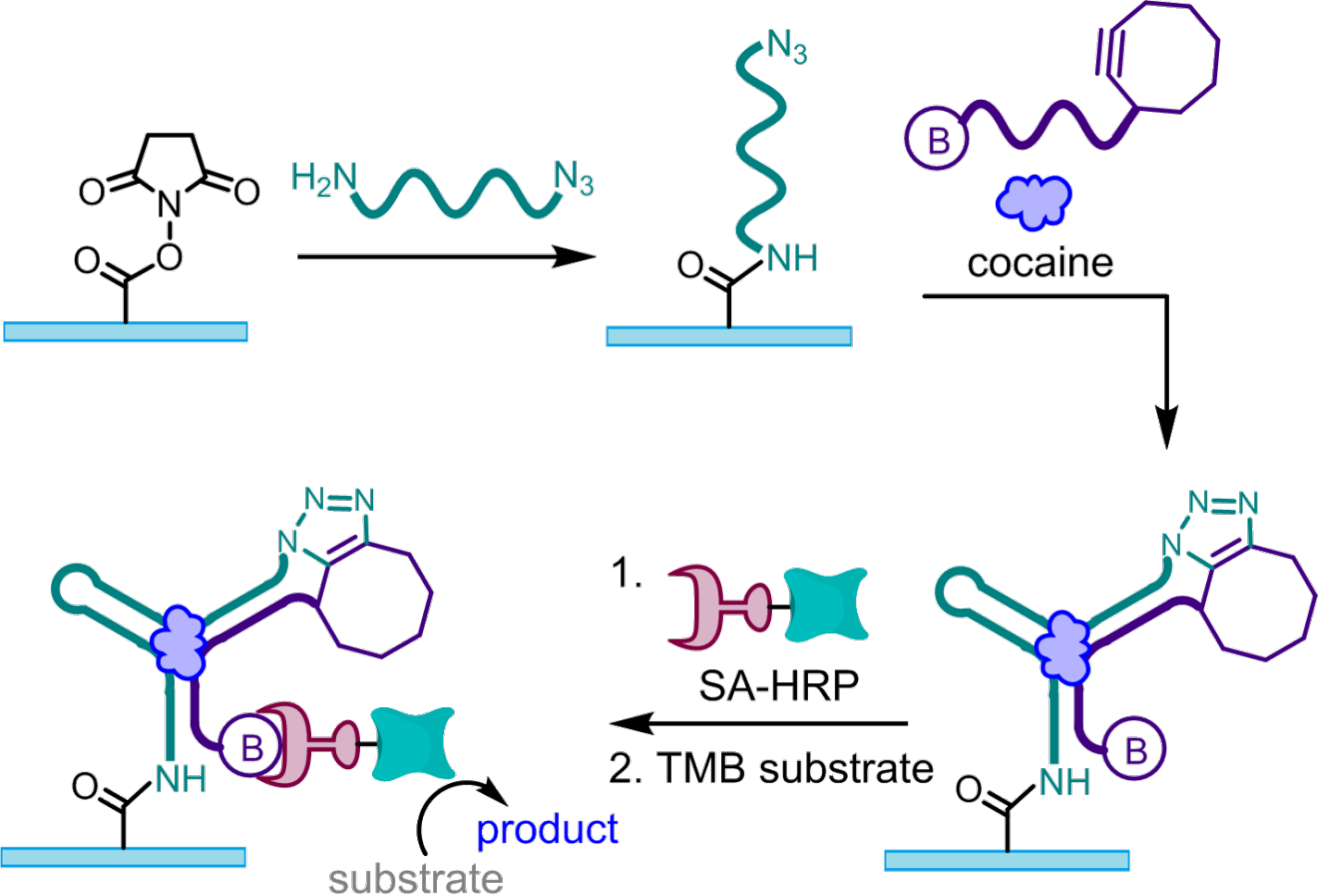
Split aptamer ligation can be used to construct an enzyme-linked assay for small-molecule detection. Conversion of the small molecule signal into a dose-dependent covalent ligation event allows signal to be produced even if target binding is lost. Reprinted with permission from [13]. Copyright (2012) American Chemical Society.

Our initial proof of concept experiments demonstrated that we could use our split aptamer ligation method to measure the concentration of a small-molecule target, but doing so required analysis via gel electrophoresis. Thus, we sought to create an assay that would be capable of the high throughput needed for clinical diagnostics applications, and we were specifically drawn to the enzyme-linked immunosorbent assay (ELISA) that is the current gold standard in this field. As shown in Figure 3, we constructed our enzyme-linked assay by immobilizing one fragment of the split aptamer on the surface of a microplate, then adding the test sample along with a solution containing the other split aptamer fragment functionalized with a biotin. In the first step of the assay, the concentration of the target molecule is transduced into a dose-dependent ligation of the split aptamer, which then allows for pull-down of a streptavidin-functionalized reporter enzyme to provide a colorimetric output [13]. This assay format highlighted the utility of our covalent trapping approach, as enzyme-linked assays typically require multiple washing steps, and loss of target binding during these steps results in loss of signal. In contrast, our method enables target binding events to be “remembered” through covalent trapping of the assembled split aptamer. Thus, the ability to generate target-dependent signal is preserved even if target binding is lost in the washing steps.

**Figure 4:**
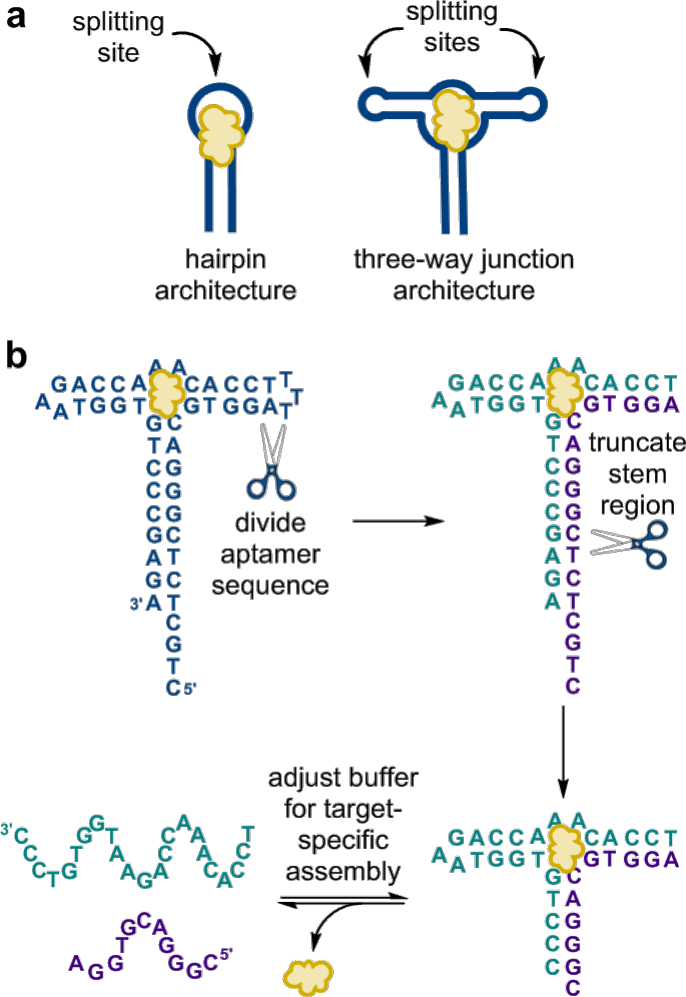
(a) In contrast to the more typical hairpin aptamer structure, we hypothesized that the three-way junction would provide a privileged architecture for generating split aptamers. (b) Split aptamers can be engineered by dividing a three-way junction aptamer, then systematically truncating the stem region to fine tune the thermodynamics of assembly. Adapted with permission from [16]. Copyright (2013) American Chemical Society.

At this point, we were excited by our successes in the development of small-molecule detection assays, but we recognized that making practical use of these assays would require DNA split aptamers for other small-molecule targets. We were initially surprised to find that while there were over 100 DNA aptamers for small-molecule targets [14], at the time, only two of these had been successfully converted to split aptamers [5–6]. We reasoned that this dearth of split aptamers was a result of the fact that many aptamer structures cannot be split without compromising substrate binding. To overcome this challenge, we hypothesized that the three-way junction architecture of the cocaine aptamer could be a privileged structure for the engineering of aptamers into split aptamers, as it offers two putative splitting sites that are distant from the typical target binding site (Figure 4a). Excitingly, Stojanovic and co-workers had recently demonstrated that SELEX could be carried out using a structurally biased library to generate aptamers having the necessary three-way junction architecture [15]. Using our insights regarding the thermodynamics of split aptamer assembly, we developed a method for rapidly and reliably converting these three-way junction aptamers into split aptamers (Figure 4b). We first divided the sequence by removing one of the loop regions, and then systematically truncated the base-pairing stems until we achieved the desired assembly properties. As predicted by our thermodynamic model, we found that split aptamer sequences having fewer base pairs could function in high ionic strength solutions, while sequences having more base pairs functioned better in lower ionic strength solutions [16]. Importantly, our success in this endeavor was again closely tied to the insights we gained from our initial unexpected results in our split aptamer ligation experiments. Looking to the future, we expect that the ability to reliably generate split aptamers for new targets of interest will greatly expand the utility of this class of recognition elements.

In parallel with our efforts aimed at small-molecule detection, my lab was also intrigued by the challenging task of measuring small-molecule enantiopurity, as this is a key factor in the synthesis of pharmaceutical intermediates and other high-value chemicals. Enantiopurity can be measured by chiral chromatographic methods, but this process is limited to a few thousand samples per day [17]. In contrast, fluorescence based methods have potential to provide throughput on the order of 10^5^–10^6^ samples per day [18]. We envisioned that aptamers could serve as powerful recognition elements for fluorescence-based high-throughput enantiopurity measurement, and the first key element to our approach was the concept of reciprocal chiral substrate selectivity. According to this principle, aptamers having the same sequence, but synthesized from opposite enantiomers of DNA, will bind to opposite enantiomers of a target molecule with identical affinity and selectivity [19]. The second key element to our approach was the ability of DNA structure-switching biosensors to transduce the presence of a target molecule into a dose-dependent fluorescence output [20]. In this sensor format, a short quencher-labeled complementary strand is hybridized to the fluorophore-labeled aptamer, and equilibrium favors duplex formation in the absence of the target. However, addition of the target molecule shifts this equilibrium to favor displacement of the complementary strand, thus generating the dose-dependent signal.

**Figure 5:**
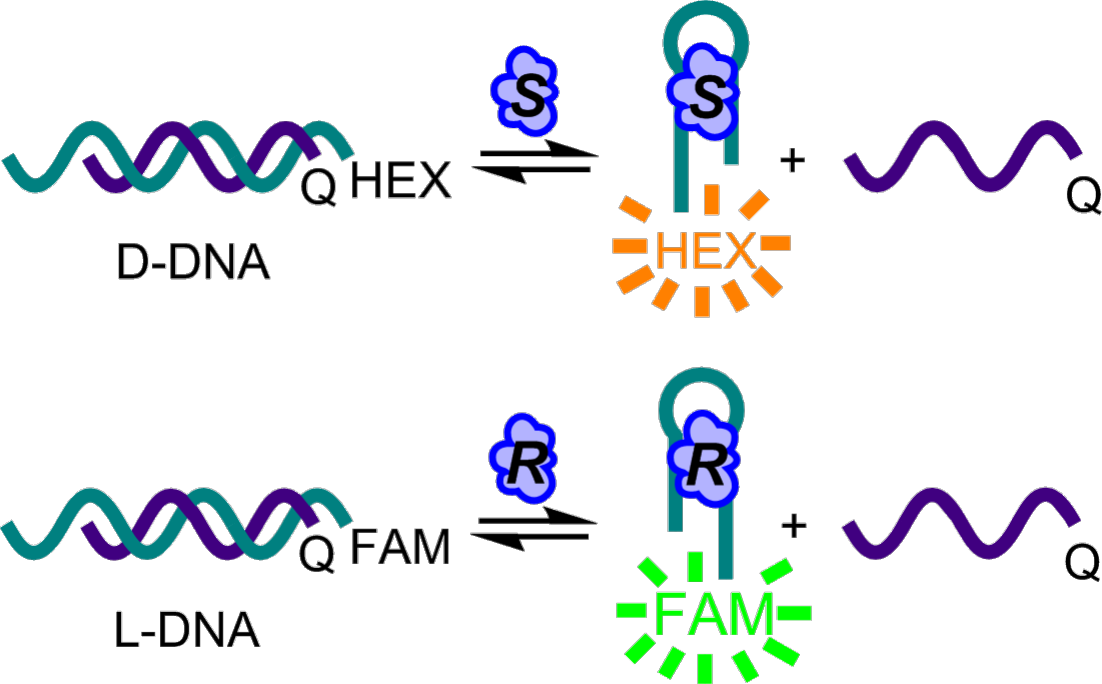
Structure switching biosensors convert the presence of a target into a dose-dependent fluorescence signal, and construction of biosensors from opposite enantiomers of DNA enables rapid measurement of enantiopurity using two-color fluorescence. Adapted with permission from [4]. Copyright (2015) American Chemical Society.

Using the previously reported structure-switching biosensor for L-tyrosinamide (L-Tym) [21], we synthesized both the L- and D-DNA sequences, but labeled these enantiomeric biosensors with orthogonal fluorophores (Figure 5). In our initial experiments, we utilized fluorescein (FAM) and cyanine 3 (Cy3), however, we observed that the difference in fluorophore structure resulted in an approximately two-fold difference in the equilibrium constants for the sensors. This was surprising, as the fluorophores are small molecules attached to the termini of much larger DNA molecules. However, we found this lesson very informative, as it showed that dyes and other functional groups that we frequently append to DNA are not as innocuous as we assume them to be. Rather, they can have a dramatic impact on the assembly properties of the DNA sequences. In our experiments, this was particularly noticeable, as the structure-switching sensors are tuned to have an equilibrium near unity, which allows small amounts of the target to trigger displacement. Thus, in these systems, subtle changes to the energetics of DNA assembly can have rather large effects on the position of equilibrium. We were fortunately able to overcome this challenge by replacing the Cy3 with a hexachlorofluorescein (HEX). HEX and FAM are spectrally orthogonal, but have similar chemical structures and electrostatic properties, and we found that they provided enantiomeric sensors having nearly identical equilibrium constants. To test our enantiopurity analysis method, the two biosensors were incubated together with varying ratios of L- and D-Tym to construct calibration curves relating the observed fluorescence output to concentration for each enantiomer. Comparison of our calculated versus actual % L-Tym for these measurements revealed a high level of both accuracy and precision, and we were also able to demonstrate the use of our sensors to accurately monitor yield and enantiopurity in chemical reactions [4]. In the context of this project, our unexpected observation regarding the impact of dyes on DNA assembly was something that we merely needed to overcome. However, this experience provided us with an improved understanding of the function of structure-switching sensors, which has proven critical to our current experiments aimed at rapidly generating these sensors for new small-molecule targets of interest.

**Figure 6:**
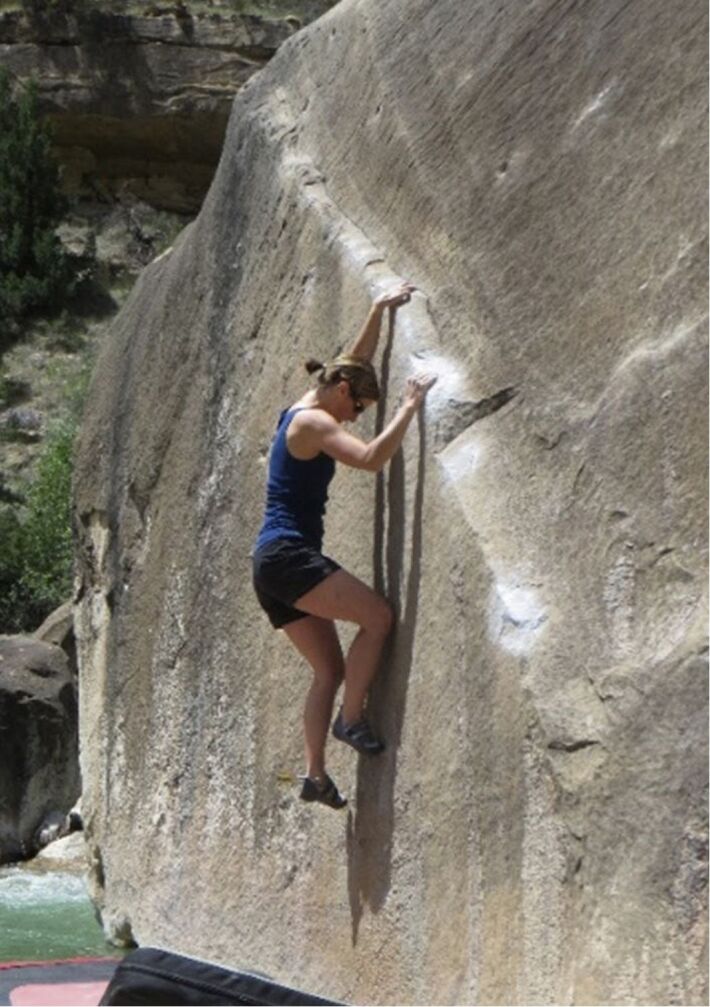
The author intensely focused on climbing the boulder problem “The Angler” during a research group outing to Joe’s Valley, UT. Photo credit: Zhesen Tan.

While I am extremely passionate about science, I find that I am most creative and happy when I can occasionally escape to pursue other hobbies. Living in Salt Lake City, I joke that my hobbies are the “Utah usual,” which includes rock climbing, road and mountain biking, snowboarding, and hiking. Among these, rock climbing, and specifically bouldering, is my favorite, as there is something special about the movement and flow of a good boulder problem. And, the intense focus required in bouldering to elucidate these complex sequences of movements is one of the few things that can completely wipe my brain clear of the stresses of deadlines and funding (Figure 6). I also very much enjoy spending time with my husband and two sons, whether we are traveling, enjoying outdoor activities, or just playing with Lego blocks.

## Conclusion

Looking toward to the future of aptamer-based sensors, I feel that there is still much to learn about the thermodynamics and kinetics of sensor assembly and target recognition. Inevitably, many of these discoveries will be made much like those I’ve highlighted above – through experiments that initially did not go as we had planned. However, I am also very thankful for the researchers who are intentionally delving into these questions and uncovering new insights on a regular basis. Understanding these principles is the key to not only designing better sensors, but also to making sure that we are generating the best possible recognition elements when we set out to select for new aptamers. In surveying the landscape of applications for DNA-based sensors, I am most excited about recent progress in the use of aptamers inside of living cells, as there is a wealth of information that can be gained regarding the concentrations and flux of small molecules within these dynamic and complex environments. Additionally, the ability to fluorescently monitor targets such as small molecules inside of living cells could prove to be valuable for synthetic biology applications such as metabolic engineering. Bringing these technologies to the point that they are routine and broadly applicable will clearly involve significant advances in molecular and cellular biology. However, contributions from supramolecular chemistry will also be critical, as these will provide the key to understanding and engineering the complex molecular architectures of aptamer-based sensors.
